# Scalable Indoor Localization via Mobile Crowdsourcing and Gaussian Process

**DOI:** 10.3390/s16030381

**Published:** 2016-03-16

**Authors:** Qiang Chang, Qun Li, Zesen Shi, Wei Chen, Weiping Wang

**Affiliations:** College of Information Systems and Management, National University of Defense Technology, Changsha 410073, China; changqiang@nudt.edu.cn (Q.C.); nudtshizesen@126.com (Z.S.); weichen@nudt.edu.cn (W.C.); wang.wp2010@gmail.com (W.W.)

**Keywords:** WLAN, indoor localization, radio map, mobile crowdsourcing, gaussian process, Bayesian algorithm

## Abstract

Indoor localization using Received Signal Strength Indication (RSSI) fingerprinting has been extensively studied for decades. The positioning accuracy is highly dependent on the density of the signal database. In areas without calibration data, however, this algorithm breaks down. Building and updating a dense signal database is labor intensive, expensive, and even impossible in some areas. Researchers are continually searching for better algorithms to create and update dense databases more efficiently. In this paper, we propose a scalable indoor positioning algorithm that works both in surveyed and unsurveyed areas. We first propose Minimum Inverse Distance (MID) algorithm to build a virtual database with uniformly distributed virtual Reference Points (RP). The area covered by the virtual RPs can be larger than the surveyed area. A Local Gaussian Process (LGP) is then applied to estimate the virtual RPs’ RSSI values based on the crowdsourced training data. Finally, we improve the Bayesian algorithm to estimate the user’s location using the virtual database. All the parameters are optimized by simulations, and the new algorithm is tested on real-case scenarios. The results show that the new algorithm improves the accuracy by 25.5% in the surveyed area, with an average positioning error below 2.2 m for 80% of the cases. Moreover, the proposed algorithm can localize the users in the neighboring unsurveyed area.

## 1. Introduction

The difficulty of determining the location of mobile users within buildings has been extensively studied for decades, due to potential applications in the mobile networking environment [1]. With the wide availability of 802.11 WLAN networks, wireless localization using Received Signal Strength Indication (RSSI) fingerprinting [2] has attracted a lot of attention.

Fingerprint indoor positioning consists of two phases: training and localization [3]. During the training phase, a database of location-fingerprint mapping is constructed. In the localization phase, the users send location queries with the current RSS fingerprints to the location server; the server then retrieves the signal database and returns the matched locations.

The accuracy of fingerprinting techniques is highly dependent on the density of the signal database. Building and maintaining a high-density database are not easy, however, for two reasons.

Firstly, building a high-density fingerprint database is labor intensive, expensive, and even impossible in some cases. Taking a 50 m × 50 m floor as an example, if we want to build a fingerprint database with a 1 m sample distance, we would have to collect 2500 samples. For each sample, we need to measure several times to get reliable results. Sometime it is impossible to collect signal fingerprints from certain locations, because of the complex local environment.

Secondly, maintaining a large signal database is expensive. As the environment changes over time due to furniture or signal sources being moved, the fingerprints diverge from those in the database. This means that the entire area needs to be re-surveyed in order to update the database. As indoor environments often change, the database would require frequent updates, which would be time-consuming and expensive.

Even though fingerprint indoor localization has many advantages, and some commercial products have been developed on this technology, such as Google Maps [4], WiFiSlam [5], and so on, challenges still exist in its application. In area without calibration data, however, this algorithm breaks down. Real-world deployment of such positioning systems often suffers the problem of sparsely available signal data. For example, Google has collected floor plans for over 10,000 locations. However, only a few of these radio maps are available for positioning.

For these problems in fingerprinting, researchers are continually searching for better algorithms to create and update dense databases more efficiently. The popularization of smartphones makes mobile crowdsourcing fingerprint localization more practical. However, designing a sustainable incentive mechanism of crowdsourcing remains a challenge. In this paper, we propose a scalable indoor positioning algorithm via mobile crowdsourcing and Gaussian Process. The basic idea behind our proposed algorithm is simple: we first propose a Minimum Inverse Distance (MID) algorithm to build a virtual database with uniformly distributed virtual Reference Points (RP). The area covered by the virtual RPs can be larger than the surveyed area. A Local Gaussian Process (LGP) is then applied to estimate the virtual RP’s RSSI values based on the crowdsourced training data. Finally, we improve the Bayesian algorithm to estimate the user’s location using the virtual database. All the parameters are optimized by simulations, and the new algorithm is tested on real-case scenarios.

In summary, we make the following major contributions:

(1) We propose a MID algorithm to build a virtual database with uniformly distributed virtual RPs. The area covered by the virtual RPs can be larger than the surveyed area.
(2) The Local Gaussian Process (LGP) is applied to estimate the virtual RPs’ RSSI values based on the crowdsourced training data.
(3) Bayesian algorithm is improved to estimate the user’s location using the virtual database.
(4) We optimize all the parameters in the proposed algorithm by simulations.
(5) An Android app is developed to test the proposed algorithm on real-case scenarios.

The rest of the paper is organized as follows: Section 2 discusses related work on building and maintaining a dense fingerprint database. Section 3 describes the details of the proposed algorithm. Section 4 optimizes the parameters and evaluates the positioning algorithm, and Section 5 concludes the paper.

## 2. Related Works

For the challenges of fingerprint positioning, much time and effort has been put into building and maintaining a dense fingerprint database with less effort [6]. Except for point by point measurement, there are mainly five ways to construct and maintain a fingerprint database.

The first is crowdsourcing [7,8,9,10,11]. The users are also database constructors. The database is updated with the most recently measured RSS uploaded by the users [12,13]. However, designing a sustainable incentive mechanism of crowdsourcing remains a challenge [14].

The second method is building the database with mathematical models. The most widely used model is the Log-Distance Path Loss (LDPL) [15,16,17,18] model. However, the indoor environments are so complex that no simple mathematical model exists to accurately predict the RSS values. Practically, LDPL only gives good results close to the AP.

Ray-Tracing [19,20,21] is the third method. However, for accurate ray tracing, you need a very detailed description of the environment such that all the reflections that eventually characterize the received signal can be simulated. Furthermore, this approach is very computationally demanding. Because of these reasons, it is only viable for small setups.

The fourth method is Simultaneous Localization and Mapping (SLAM) [22,23]. In SLAM, the database is populated on the fly, provided that the users are equipped with a receiver and an IMU. In general, the accuracy of positioning with this technique is lower because the database is less accurate.

The fifth way is the combination of the previous methods. A few RPs cover a large range of area and are collected or generated by the previous method. The remaining RP RSS values are estimated mathematically. Linear and exponential taper functions are used by [24]; the Motley–Keenan model [25] and a semi-supervised manifold learning technique [26] are also used by researchers [27]. Liqun Li propose Modellet [28] to approximate the actual radio map by unifying model-based and fingerprint-based approaches. However, their algorithm only works for nodes near the Access Points (APs). Gaussian Process (GP) [29] is a non-parametric model that estimates Gaussian distribution over functions based on the training data [30]. GP is suitable for estimating RSS values [31]. However, GP is computational consuming, meaning it is not a satisfactory method of generating a large scale area’s signal strength.

There are also some other researchers that improved fingerprinting performance by introducing new sensors. For example, IMU [32,33], barometer [34], and so on. However, extra running sensors not only consume more battery, but also bring in new errors. Some such algorithms required the sensors to keep running even if the user does not need the positioning service, which is not suitable for our daily use.

Our study is motivated by these pioneer works, but we approached the problem from a different angle and mainly focus on a scalable indoor positioning algorithm that works both in surveyed and unsurveyed areas. We propose a novel algorithm to create WLAN radio map by mobile crowdsourcing and Gaussian Process. We first propose a Minimum Inverse Distance (MID) algorithm to build a virtual database with uniformly distributed virtual Reference Points (RPs). A Local Gaussian Process (LGP) is then applied to estimate the virtual RPs’ RSSI values based on the crowdsourced training data. Finally, we improve the Bayesian algorithm to estimate the user’s location using the virtual database. We didn’t use the crowdsourced data for positioning directly, and we didn’t introduce other sensors to improve the performance either.

## 3. Materials and Methods

### 3.1. Problem Setting and Algorithm Overview

We only concentrate on the 2D positioning problem in this paper. Assuming the target area is denoted as *P*, the area of *P* is $S(m^{2})$. There are *a* Wi-Fi APs in the target area.

In crowdsourced fingerprint positioning, the radio map is created by users. The RSS values in different RPs from different signal sources are measured and uploaded to the database together with the coordinates. The coordinates come from another positioning system, such as GNSS, or specified by the users.

Assuming we have built a signal database $DB^{Crowd}$ by crowdsourcing, and there are *n* RPs in $DB^{Crowd}$. The RPs are denoted as $RP_{i}=\left\{p_{i},F_{i},\sigma_{i}\right\},i=1,2,\cdots ,n$, where $p_{i}=\left(x_{i},y_{i}\right)$ and $F_{i}=\left\{\left(Mac_{j},RSS_{i,j}\right),j=1,2,\cdots ,a\right\}$. $\sigma_{i}$ is the measurement variance. The density of $DB^{Crowd}$ is denoted as $\rho^{Crowd}=n/S$. The problem in fingerprint localization is estimating the user’s current coordinate $p_{t}$ at time *t* based on the measurement $F_{t}$ and the database $DB^{Crowd}$.

The density of $DB^{Crowd}$ will be different from region to region. As a result, the positioning accuracy will be different for the area. If we want a continuous positioning performance, we need a database with uniformly distributed reference points. There also might be certain areas without RPs, e.g., because of the complex local environment or not covered due to some other reasons. This method breaks down.

**Figure 1:**
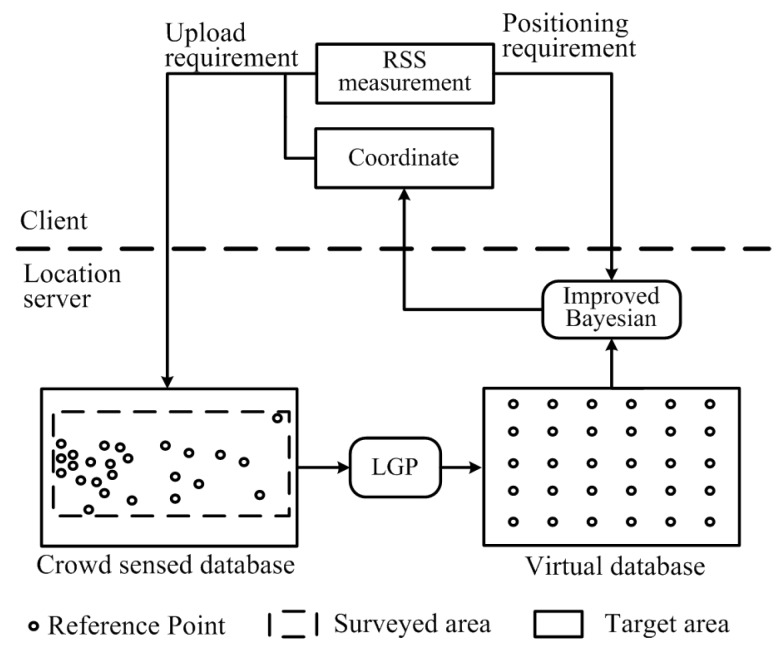
Framework of the proposed algorithm. RSS: Received Signal Strength; LGP: Local Gaussian Process.

In this paper, we propose a novel algorithm to create a virtual WLAN radio map by mobile crowdsourcing and Gaussian Process for scalable indoor positioning. This virtual radio map is denoted as $DB^{\left(v\right)}$. $DB^{\left(v\right)}$ contains *m* RPs, so that the density of the virtual database is $\rho^{\left(v\right)}=m/S$. The $i^{th}$ RP in $DB_{\left(v\right)}$ is $RP_{i}^{\left(v\right)}$. $RP_{i}^{\left(v\right)}=\left\{p_{i}^{\left(v\right)},F_{i}^{\left(v\right)},\sigma_{i}^{\left(v\right)}\right\}$, where $p_{i}^{\left(v\right)}=\left(x_{i}^{\left(v\right)},y_{i}^{\left(v\right)}\right)$ and $F_{i}^{\left(v\right)}=\left\{\left(Mac_{j},RSS_{i,j}^{\left(v\right)}\right),j=1,2,\cdots ,a\right\}$. $RSS_{i,j}^{\left(v\right)}$ is AP $j^{\prime }s$ RSSI measured at RP *i*, and $\sigma_{i}^{\left(v\right)}$ is the variance of the measurement. $RSS_{i,j}^{\left(v\right)}$ is estimated using our proposed Local Gaussian Process (LGP) based on $DB^{Crowd}$. The user makes use of $DB^{\left(v\right)}$ for positioning. Figure 1 shows the framework of the proposed algorithm.

After collecting RSS values from surrounding APs, if the user gets the current coordinate by other methods, he can upload the fingerprint containing the coordinate and the RSS values to the server. The server will add the fingerprint to $DB^{Crowd}$, and update $DB^{\left(v\right)}$ using LGP. If the user wants to estimate current location, he can send the positioning requirement, including the RSS measurement, to the server. The server will estimate the coordinate using the proposed Bayesian algorithm based on $DB^{\left(v\right)}$ and then send the result to the user.

In the next section, we first built a dense virtual database by introducing uniformly distributed virtual RPs in the area, and then we propose the Local Gaussian Process (LGP) to estimate the virtual RPs’ RSSI values and the variance. We improved the Bayesian algorithm to estimate the user’s location using the virtual database.

### 3.2. Building the Dense Virtual Database

As stated earlier, the fingerprints in the virtual database should be selected as uniformly as possible over the target area. *m* is the number of RPs in $DB^{\left(v\right)}$. However, for general values of *m*, it is not straightforward to uniformly distribute the RPs over the area. Therefore, we propose a low-complexity algorithm to select the positions of the RPs: the Minimum Inverse Distance (MID) algorithm. In this algorithm, the selection of the positions of the RPs is based on a virtual sample database $DB^{Sample}$, which is constructed by placing a square grid in the target area with grid size *λ*, where the positions of the virtual RPs are selected as the corners of the squares in the grid. Assuming the target area has size $x_{max}\times y_{max}$, the number of virtual positions equals $\left\lfloor x_{max}/\lambda \times y_{max}/\lambda \right\rfloor$. The *m* positions of the RPs for virtual database $DB^{\left(v\right)}$ are selected out of the sample database $DB^{Sample}$. We initialize the algorithm by randomly choosing one virtual position $RP_{e}$ from $DB^{Sample}$: $DB^{\left(v\right)}=\left\{RP_{e}\right\}$. The other m-1 positions are picked from the virtual database $DB^{Sample}$ based on the measure in Equation (1):

(1) $$Dis_{i}=\sum_{j}\frac{1}{{\left(x_{i}-x_{j}\right)}^{2}+{\left(y_{i}-y_{j}\right)}^{2}}$$

where $\left(x_{i},y_{i}\right)$ is the coordinate of the candidate position $\in DB^{Sample}$ and $\left(x_{j},y_{j}\right)$ are the coordinates of the RP positions already present in the database $DB^{\left(v\right)}$. The virtual position that minimizes $Dis_{i}$ is selected and added to the database $DB^{\left(v\right)}$. Because the measure function $Dis_{i}$ is inversely proportional to the Euclidean distances between the candidate RP and the RPs in the database $DB^{\left(v\right)}$, candidate positions that are far from the already selected RP positions are favored, while candidate positions near already selected RP positions are filtered out. As a result, the distances between the RPs will be maximized and the RPs in $DB^{\left(v\right)}$ will be distributed uniformly and expand to the very edges of the target area. We call this algorithm as Minimum Inverse Distance (MID) algorithm. Details of MID are shown in Algorithm 1.

**Algorithm 1****Require** the target area *P*, the distance *λ* between neighbor virtual RPs in $DB^{sample}$ the number *m* of RPs we want to select.**Ensure** select RPs every *λ* meters in *P* to build $DB^{sample}$**Ensure** randomly select $RP_{e}$ from $DB^{sample}$, $DB^{\left(v\right)}=\left\{RP_{e}\right\}$  **While**($|DB^{\left(v\right)}|\neq m$)    **For all**($RP_{i}\subset DB^{sample}$)      Calculate $Dis_{i}$ using Equation (1)    **End all**    $RP=\arg\min_{RP_{i}\in DB^{sample}}Dis_{i}$    $DB^{\left(v\right)}\leftarrow RP$  **EndWhile**

**Figure 2:**
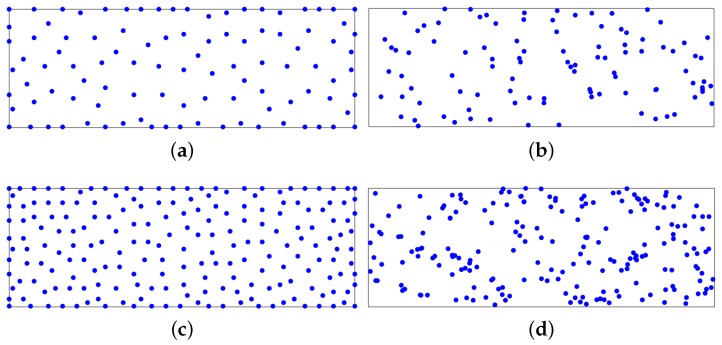
Positions of the Reference Points (RPs) (**a**) Minimum Inverse Distance (MID) , m=100; (**b**) randomly, m=100; (**c**) MID, m=200; (**d**) randomly, m=200.

To illustrate MID, we consider an area *P* of 19.5 m × 48.5 m and λ=0.5 m. Figure 2 shows the positions of the RPs in $DB^{\left(v\right)}$ when m=100 and 200 RPs are selected out of the virtual sample database $DB^{Sample}$. Further, Figure 2 shows the positions of the RPs when the RPs are selected randomly from $DB^{Sample}$.

As can be observed, the proposed algorithm is able to select any number of RPs spatially uniform over the target area.

After the positions of the RPs in database $DB^{\left(v\right)}$ are selected with MID, the RSS values and the variance for these RPs need to be determined. To this end, we compare the positions of the RPs in $DB^{\left(v\right)}$ with those in $DB^{Crowd}$. Whenever one or more RPs in $DB^{Crowd}$ are within a distance *ε* of a RP $RP_{i}$ in $DB^{Crowd}$, we will replace the position of the RP in $DB^{\left(v\right)}$ with the position of the nearest RP in $DB^{Crowd}$, together with its RSS values and the variance on the measurement. If no RPs in $DB^{Crowd}$ are within a distance *ε* of a RP $RP_{i}$ in $DB^{Crowd}$, the Local Gaussian Process (LGP) algorithm will be used to estimate the RSS values and their variance in $RP_{i}$.

The resulting virtual database $DB^{\left(v\right)}$ is determined by three parameters: the number *m* of RPs in $DB^{\left(v\right)}$, the distance *λ* between RPs in the virtual sample database, and the radius *ε* within which nearby training RPs are looked for. The number *m* of RPs is defined by the positioning accuracy. The distance *λ* determines not only the spatial uniformity of the resulting RPs, but also the complexity of the algorithm: by reducing *λ*, the RPs will be placed more uniformly over the area *P*, but the complexity of MID increases as the number of virtual RPs to be searched increases in an inverse proportion to the quad-rate of *λ*. Finally, the radius *ε* will also have an influence on the positioning accuracy. When the radius is small, the resulting database $DB^{\left(v\right)}$ will have a more uniform placement of RPs, but the probability of finding a nearby training RP decreases, such that the RSS of more RPs needs to be determined using the LGP algorithm. On the other hand, when the selected radius is large, the resulting database $DB^{\left(v\right)}$ will be less spatially uniform, but more training RPs will be present in $DB^{\left(v\right)}$. In Section 4, we will optimize them before positioning.

### 3.3. Local Gaussian Process

The Local Gaussian Process (LGP) algorithm is used to reduce the computational complexity of the Gaussian Process (GP) algorithm, which is used to predict unknown RSS values at positions that are not in the training database [29]. In this section, we first review the GP algorithm. This algorithm starts from the property that RSS values at surrounding positions are correlated. Because of this correlation, it is possible to describe the RSS at positions where the RSS is not known as function of the RSS at positions where the RSS value is measured. The GP algorithm uses the Gaussian kernel to describe this correlation. As a result, the correlation matrix between the noisy RSS values $RSS_{i}$ at positions $c_{i}=\left\{x_{i},y_{i}\right\}$, i=1,…,n, measured during the training phase, can be written as:

(2) covρ=Q+S

where $\rho (i)=RSS_{i}$, $Q_{i,j}=k\left(c_{i},c_{j}\right)$, and $S=diag\{\sigma_{i}^{2}\}$ is the diagonal matrix of the variances of the measured RSS values $RSS_{i}$. Further, $k(c_{i},c_{j})$ is the Gaussian kernel function:

(3) $$k\left(c_{i},c_{j}\right)=\sigma_{f}^{2}\exp(-\frac{1}{2l^{2}}\left|\right|c_{i}-c_{j}{\left|\right|}^{2})$$

where $\sigma_{f}^{2}$ and *l* are the signal variance and length scale, respectively, determining the correlation with the RSS values at surrounding positions. The parameters $\sigma_{f}^{2}$, and *l* can be estimated using hyper-parameter estimation [5]. This covariance matrix can be used to predict the RSS value $RSS_{*}$ at an arbitrary position $c_{*}=\left\{x_{*},y_{*}\right\}$. The posterior distribution of the RSS value at any position is modeled as a Gaussian random variable, *i.e.*, $\left(RSS_{*}|c_{*}\right)=N\left(RSS_{*};\mu_{*},\sigma_{*}^{2}\right)$, where $\mu_{*}$ and $\sigma_{*}^{2}$ are given by:

(4) $$\mu_{*}=k_{*}^{T}{\left(Q+S\right)}^{-1}\rho$$

(5) $$\sigma_{*}^{2}=k\left(c_{*},c_{*}\right)-k_{*}^{T}{\left(Q+S\right)}^{-1}k_{*}+\sigma_{n}^{2}$$

with $\sigma_{n}^{2}$ is the measurement variance, $k_{*}\left(i\right)=k\left(c_{*},c_{i}\right)$, i=1,…,n. The estimate of the RSS value at position $c_{*}=\left\{x_{*},y_{*}\right\}$ equals $RSS_{*}=\mu_{*}$ and the uncertainty on the estimated RSS is $\sigma_{*}^{2}$.

For a large area containing several hundred RPs, computing the RSS values with Equations (4) and Equation (5) is computationally demanding because of the inversion of the large covariance matrix Equation (2). However, in an indoor environment, we may assume that RPs at a large distance from the position where we want to estimate the RSS value are blocked by several walls and other objects. Hence, the covariance k(·,·) between the RSS values of those far away RPs and the RSS values at the considered position will be approximately zero. As a result, it is a reasonable assumption that only training RPs close to the considered position will contribute to the RSS value at the considered position. The LGP algorithm restricts the training RPs that contribute to the RSS value at position $c_{*}$ to a training set $TS_{*}$, setting $k(x_{*},x_{i})=0$ if $x_{i}\notin TS_{*}$. Assuming the number of RPs in $TS_{*}$ equals *L*, the LGP algorithm simplifies Equations (4) and (5) by only considering the *L* nearest to RPs. That is, $k_{*}$ and *ρ* reduce to a L×1 vector, and covρ Equation (2) to a L×L matrix. Compared to the complexity $O(n^{3})$ when all *n* RPs in the training database are used, the LGP algorithm has complexity O(nL) to select the *L* nearest RPs and $O(L^{3})$ to invert the reduced-size covariance matrix Equation (2).

**Figure 3:**
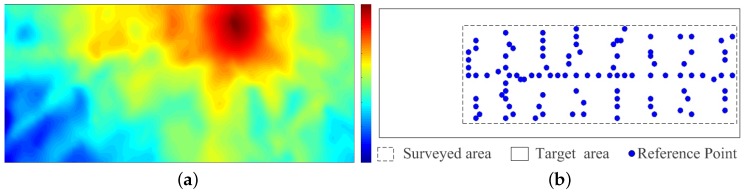
True radio map and the distribution of RPs. (**a**) True Radio map of the whole target area; (**b**) Distribution of Reference Points.

To illustrate the LGP algorithm, we consider the RSS radio map of a WiFi access point in an indoor environment. The true radio map is created using the WinProp tool from AWE Communications [35], denoted as DB. The area is a 19.5 m × 48.5 m rectangle, containing 18 rooms in the same floor. The true radio map contains 3318 uniformly distributed RPs. We select 100 RPs from DB, which covers part of the target area. Figure 3 shows the true radio maps and the distribution of the selected RPs.

We apply the proposed LGP to create the radio map for the target area. Part of the un-surveyed areas are included.

**Figure 4:**
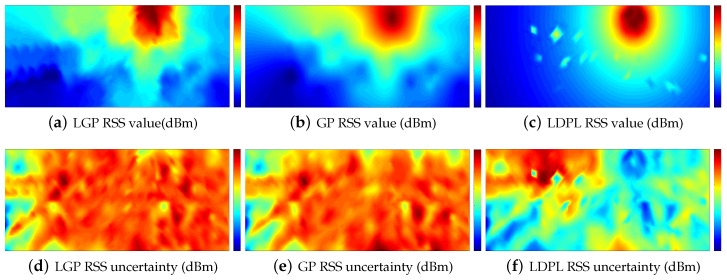
The estimated RSS values and the uncertainty. LGPL: Log-Distance Path Model.

There are some algorithms can be applied to build the radio map rapidly presented in Section 2, such as crowdsourcing, ray-tracing, SLAM, and mathematical models. We did not supply enough comparison with all of these techniques because different algorithms rely on different equipment and input. It is not straightforward to make comparisons between different algorithms in different conditions. Our study mainly focuses on the mathematical model. As a result, we only make comparisons between the widely used mathematical models, including Gaussian Process (GP) and Log-Distance Path Model (LDPL). Figure 4 is the simulation result. During the simulation, we set λ=0.5, L=4, m=800, and ε=0.5. In the Log-Distance Path Model (LDPL) [36], where the parameters of the LDPL model are estimated based on the training data, the uncertainty of RP *i* is defined as follows:

(6) $$Diff_{i}=F_{i}-{\hat{F}}_{i}$$

where ${\hat{RSS}}_{i,j}$ and $RSS_{i,j}$ are estimated and true RSS values at RP *j*, respectively.

As can be observed, the radio maps for GP and LGP are similar to the true radio map. The LDPL model, which is known to fail at positions far from the signal source, resembles the true radio map less, comparatively.

We also compute the variance over all RPs. The variance is defined in Equation (7).

(7) $$\sigma =\sqrt{\sum_{i=0}^{m}Diff_{i}^{2}/m}$$

We evaluate the average uncertainty for different areas, which are Surveyed Area (SA), Unsurveyed Area (UA) and the Target Area (TA, TA=SA∪UA). Table 1 illustrates the simulation results.

From Table 1, we can see that GP has the lowest variance in the target area, which is about 5.77 dBm, followed by LGP with an average of 5.88 dBm. The highest variance comes from LDPL, which is 7.43 dBm. In the surveyed area, all the three algorithms perform better than in the unsurveyed area. In all cases, GP performs the best over the three algorithms.

**Figure 5:**
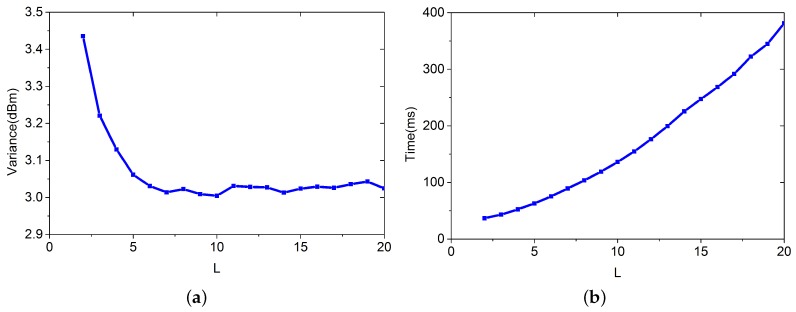
Variance and Time complexity vary with different *L*. (**a**) Variance of the estimation; (**b**) Time for building the virtual database.

*L* is the number of training RPs used for estimating the RSS values for a given virtual node. A large *L* introduces more training data, and a more accurate result is obtained. However, the time for estimating the RSS values will be increased. In this section, we explore the question of how to find a good balance between the variance and the time for building the virtual database. During the simulation, we set λ=0.5, ε=0.5, and m=800. Figure 5 shows the result.

In this simulation, *L* increases from 2 to 20. In Figure 5a, we can see that the variance decreases as *L* increases. Figure 5b shows the time complexity increase with *L*. If we want to keep a good balance between time complexity and variance, we can set L=7.

For a more accurate result, we evaluate the three algorithms with different densities of $DB^{Crowd}$.

**Figure 6:**
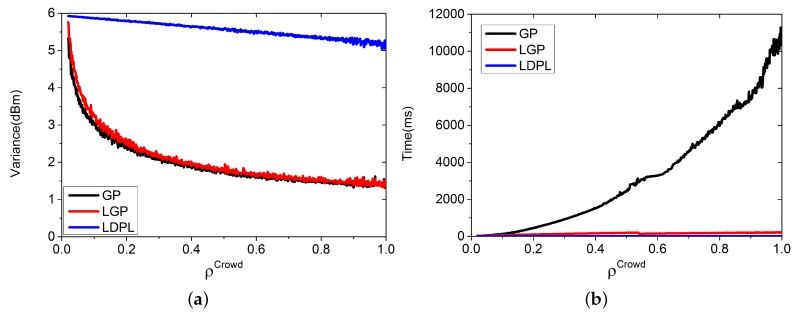
Variance and Time complexity vary with different $\rho^{Crowd}$. (**a**) Variance of the estimation; (**b**) Time for building the virtual database.

In the following simulation, we set $\rho^{Crowd}$ varying from 0.02 to 1, and the training RPs were selected randomly from DB. For each value of $\rho^{Crowd}$, we simulated 2000 times with λ=0.5, ε=0.5, m=800, and L=7. Figure 6 shows the results. Time refers to the time for building the virtual database.

In Figure 6a, GP performs the best, followed by LGP, and LDPL performs the worst. However, the differences between GP and LGP are small. In Figure 6b, LDPL has the lowest time complexity, followed by LGP and GP. In summary, LGP keeps a good balance between variance and time complexity.

### 3.4. Improved Bayesian Algorithm

Given measurement $F_{t}$ at time *t* and $DB^{\left(v\right)}$, the objective in fingerprint localization is to estimate the user’s real-time coordinate $p_{t}$ at time *t*.

The Bayesian localization algorithm is suitable for a user contribution-based localization system for mobile devices [8]. The standard Bayesian localization algorithm will calculate all the RPs’ posterior probability, and maximum them to estimate the coordinates. If the fingerprint database contains a great number of RPs, computing all the RPs’ posterior probability would be time consuming. In this paper, we first select *K* nearest RPs from the virtual database based on the metric defined in Equation (8).

(8) $$d_{t,i}=\sum_{s=1}^{a}{\left(|RSS_{t,s}-RSS_{i,s}{|}^{q}\right)}^{1/q}$$

These *K* RPs have the smallest $d_{t,s}$ among the others in $DB^{\left(v\right)}$. A standard Bayesian localization algorithm was used to estimate the user’s real-time coordinates based on the selected RPs. The posterior probability of being in one of the selected RPs’ locations is given by Equation (9):

(9) $$P\left(p_{i}|F_{t}\right)=\frac{P\left(p_{i}\right)*P\left(F_{t}|p_{i}\right)}{\sum_{j=1}^{m}P\left(p_{j}\right)*P\left(F_{t}|p_{j}\right)}$$

where P() represents the probability density function, $P(p_{i})$ is the prior probability of the user’s location, and $P(F_{t}|p_{i})$ is the likelihood of observing a set of signal strength measurements $F_{t}$ at location $p_{i}$. $p_{i}$ is assumed to be uniformly distributed.

For simplicity, we assume that each signal strength $RSS_{i,j1}$ is conditionally independent of every other $RSS_{i,j2}$ for j1≠j2. So, we have:

(10) $$P\left(F_{t}|p_{i}\right)=\prod_{j=1}^{a}P(RSS_{t,j}|p_{i}),i=1,2,\ldots ,K$$

**Figure 7:**
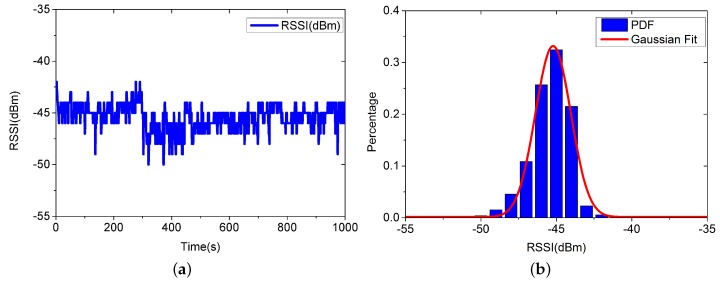
RSSI data and Gaussian Fit. (**a**) RSSI values measured at a stationary location; (**b**) PDF and Gaussian Fit.

For modeling the conditional probability $P(RSS_{t,j}|p_{i})$, we first show the measurement results from a specified AP at a stationary location in Figure 7.

Figure 7 implies that we can model the conditional probability $P(RSS_{t,j}|p_{i})$ as a Gaussian distribution:

(11) $$P\left(RSS_{t,j}|p_{i}\right)=\frac{1}{\sqrt{2\pi }\sigma }e^{-\frac{{\left(RSS_{t,j}-RSS_{i,j}\right)}^{2}}{2\sigma^{2}}}$$

The users have to estimate the RSS variance $\sigma_{n}^{2}$ before uploading the measurement. For the virtual RPs, the variance is given by Equation (5).

Finally, we estimate the user’s coordinates using Equation (12):

(12) $$p_{t}=\sum_{i=1}^{k}p_{i}\hat{P}\left(F_{t}|p_{i}\right)$$

where $\hat{P}\left(F_{t}|p_{i}\right)$ is the normalized conditional probability given by Equation (13):

(13) $$\hat{P}\left(F_{t}|p_{i}\right)=\frac{P(F_{t}|p_{i})}{\sum_{j=1}^{k}P(F_{t}|p_{j})}$$

## 4. Results and Discussion

There are some parameters in the proposed algorithm, including *λ* in MID, *ε* in LGP, *m* the number of RPs in virtual database, and *K* in the improved Bayesian. All of these parameters determine the complexity and positioning accuracy of the proposed algorithm. We first optimize these parameters by simulations, and then we evaluate the proposed algorithm by a real-case scenario experiment.

In the following section, root mean square error (RMSE) is defined in Equation (14):

(14) $$RMSE=\sqrt{\frac{\sum_{i=1}^{W}\left[\left(x_{i}-{\hat{x}}_{i}\right)+\left(x_{i}-{\hat{x}}_{i}\right)\right]}{W}}$$

where $\left(x_{i},y_{i}\right)$ and $\left({\hat{x}}_{i},{\hat{y}}_{i}\right)$ are the true and estimated coordinates of the user and *W* is the number of positioning cases.

### 4.1. Optimize the Parameters in the Algorithm

#### 4.1.1. *λ*

The distance *λ* determines not only the spatial uniformity of the resulting RPs, but also the complexity of the algorithm: by reducing *λ*, the RPs will be placed more uniformly over the area, but the complexity of MID increases as the number of virtual RPs to be searched increases in inverse proportion to the quad-rate of *λ*.

**Figure 8:**
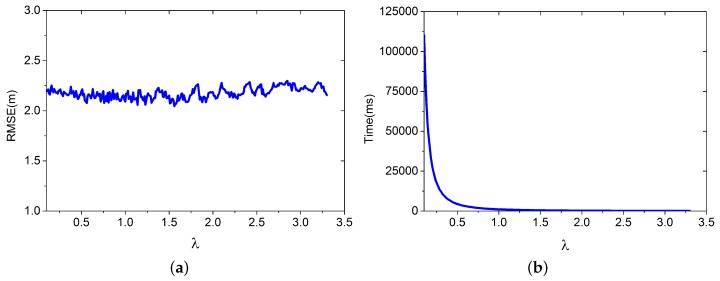
Root mean square error (RMSE) and time for building the virtual database vary with different *λ*. (**a**) RMSE.; (**b**) time for building the virtual database.

In this simulation, we build different $DB^{\left(v\right)}$ based on different $DB^{Sample}$ for positioning. The training database are randomly selected from DB, containing 80 RPs. We apply the LGP for estimating the virtual RPs’ RSS values. The improved Bayesian algorithm is applied for positioning. The other parameters are set as follows: ε=0.5, L=7, m=80, and K=3; *λ* is set to increase from 0.1 to 3.3. Results from 3000 positioning cases are shown in Figure 8.

Figure 8a shows that RMSE doesn’t change significantly with *λ*. Figure 8b shows that the time decreases when *λ* increases. The results from Figure 8 tell us that we can use as large a *λ* as possible to reduce the time for building the virtual database.

#### 4.1.2. *ε*

The radius *ε* has an influence on the positioning accuracy. When the radius is small, the resulting database $DB^{\left(v\right)}$ will have a more uniform placement of the RPs, but the probability of finding a nearby training RP decreases, such that the RSS of more RPs need to be determined using the LGP algorithm. On the other hand, when the selected radius is large, the resulting database $DB^{\left(v\right)}$ will be less spatially uniform, but more training RPs will be present in $DB^{\left(v\right)}$.

**Figure 9:**
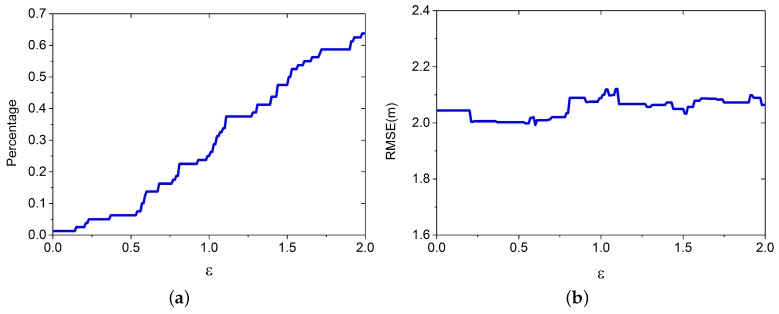
Percentage of training data and RMSE vary with different *ε*. (**a**) Percentage of training data; (**b**) RMSE.

Similar with the previous setting, we apply the LGP for estimating the virtual RPs’ RSS values, and the improved Bayesian algorithm for positioning. The other parameters are set as follows: λ=3.3, L=7, m=80, and K=3. We build $DB^{\left(v\right)}$ based on the crowdsourcing database, which contains 80 RPs and is randomly selected from DB. *ε* is set to increase from 0 to 2. Results from 3000 positioning cases are shown in Figure 9.

Figure 9a shows that the training RPs’ percentage increase as *ε* increases. Figure 9b shows that more training data doesn’t improve the performance, because $DB^{\left(v\right)}$ is less spatially uniform.

#### 4.1.3. *K*

**Figure 10:**
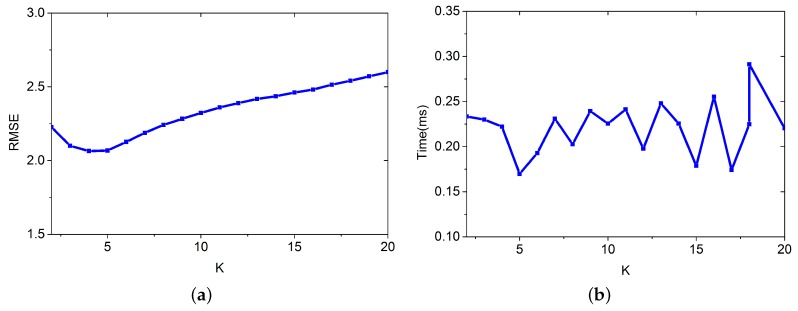
Time for positioning and RMSE vary with *K*. (**a**) RMSE; (**b**) Time for positioning.

*K* is the number of nodes used for positioning. In this simulation, we want to find the best *K* for estimation. We apply the improved Bayesian algorithm for positioning. We build $DB^{\left(v\right)}$ based on the crowdsourcing database, which contains 80 RPs and is randomly selected from DB. We set *K* to increase from 1 to 10. The other parameters are set as follows: λ=3.3, L=7, m=80, and ε=0.5. Results from 3000 positioning cases are shown in Figure 10.

The result from Figure 10a shows that using 4 or 5 RPs for positioning performs the best. And Figure 10b shows that the time for positioning is not sensitive to *K*.

#### 4.1.4. *m*

**Figure 11:**
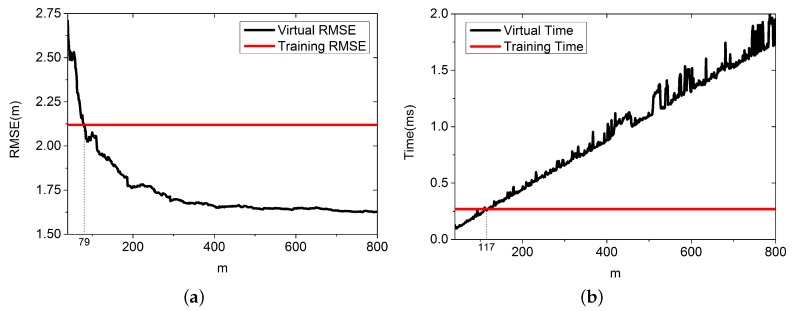
Time for positioning and RMSE with varying *m*. (**a**) RMSE; (**b**) Time for positioning.

*m* is the number of RPs in the virtual database DB. More RPs might result in a more accurate positioning result, but also needs more time for querying in the database. In this section, we want to find the best size of the virtual database. In this simulation, we set *m* to increase from 40 to 800, and the training database contains 80 randomly distributed RPs selected from DB. The other parameters are set as follows: λ=1, L=7, K=5, and ε=0.5; For each value of *m*, the results come from 3000 positioning cases. Figure 11 shows the result.

Figure 11a shows that when *m* is about the same as the number of training RPs, the two methods perform the same. However, as *m* increases, the proposed algorithm performs better. When m=800, the RMSE is 1.63 m compared with 2.12 m using the training database. The proposed algorithm improves the accuracy by about 23.2%. Figure 11b shows that when m=117, it performs the same as using training database, and the proposed algorithm needs more time for positioning.

### 4.2. Real-Case Scenario Experiment

**Figure 12:**
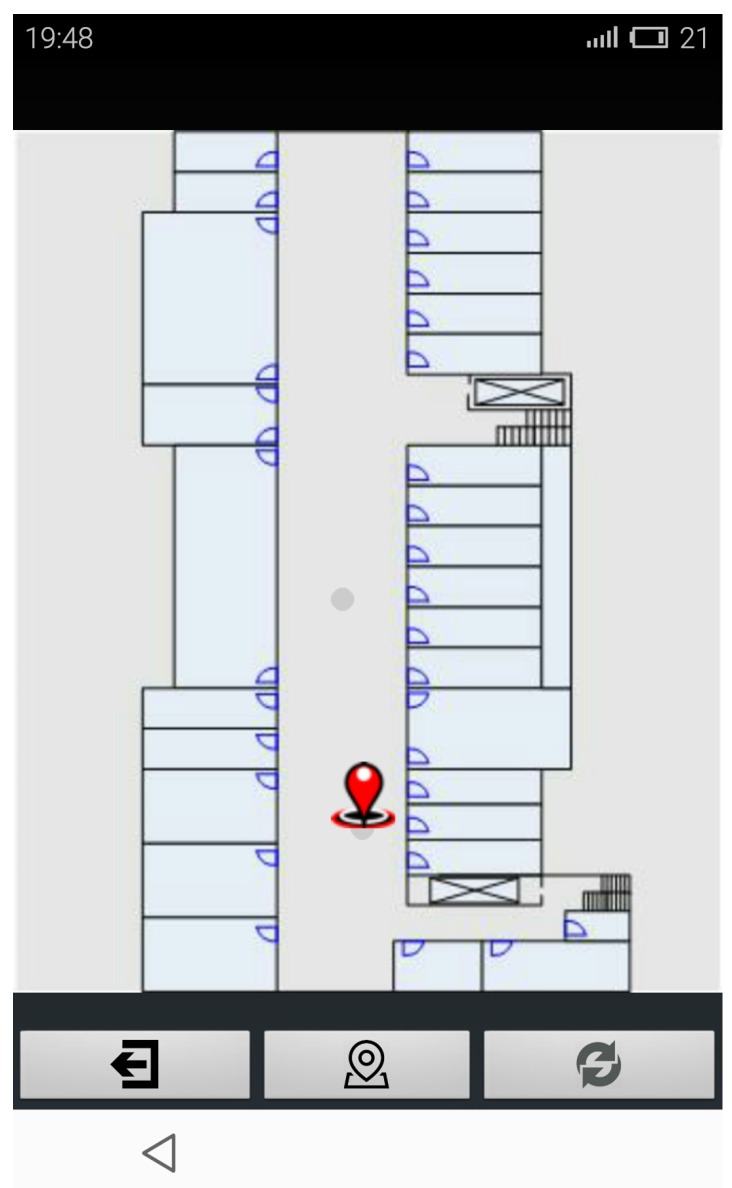
User interface of the app.

For testing the new algorithm in real world, we developed an Android app. The indoor radio map is build in a crowdsourcing way. The user can locate themselves, and they can also upload the fingerprint data to the location server. Figure 12 is the user interface of the app.

In Figure 12, the map is the floor layout of our Lab, covering an area of about 1928 m${}^{2}$. Clicking the central button will send positioning requirement. Long pressing the interface will change the map of the indoor environment. Double clicking the interface will specify a user’s current location. The user can click the right button to send the current RSS measurement and the specified coordinate to the training database.

**Figure 13:**
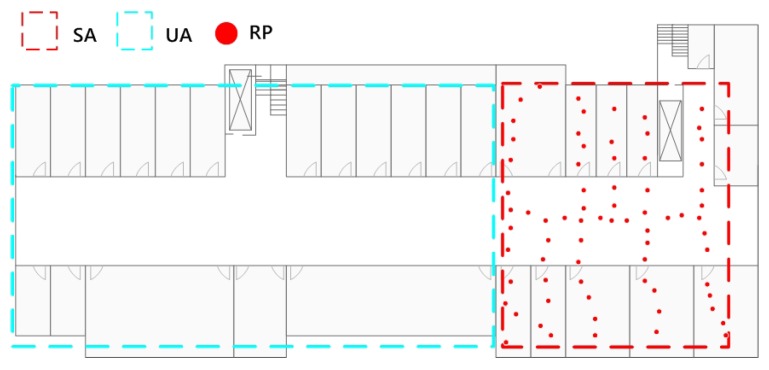
Distribution of initial data.

We first built a training database covering part of the target area. The database contains 71 RPs. We will test the new algorithm in the surveyed area and in different unsurveyed areas. Figure 13 is the distribution of the initial data.

**Figure 14:**
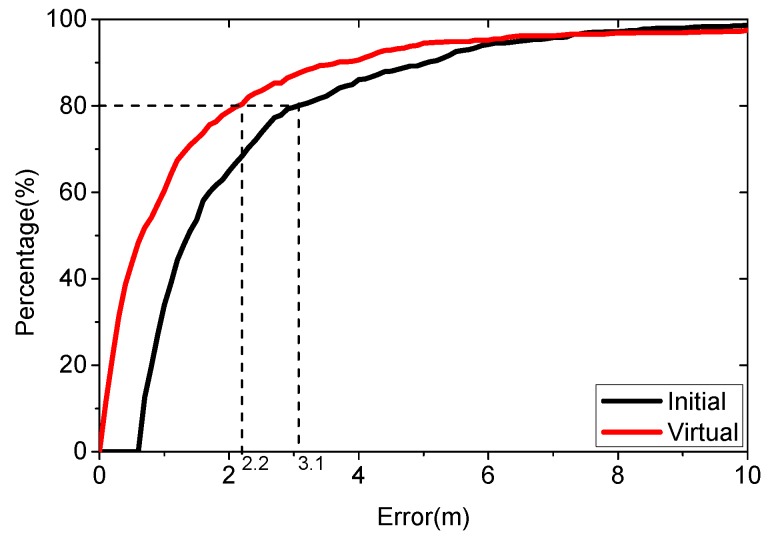
Positioning result using initial database and virtual database in the surveyed area.

We first estimate the user’s coordinate using virtual database and training database in the surveyed areas. The parameters are set as follows: λ=1, L=7, K=5, ε=0.5. The density of the virtual database is 1. Figure 14 shows the results from 3000 positioning cases.

Figure 14 shows that the proposed algorithm performs better. The average localization error is 2.47 m using the initial database, while it is 1.84 m using the virtual database. The new algorithm improves the accuracy by 25.5%, with an average positioning error below 2.2 m for 80% of the cases, while the virtual database is 3.1 m.

**Figure 15:**
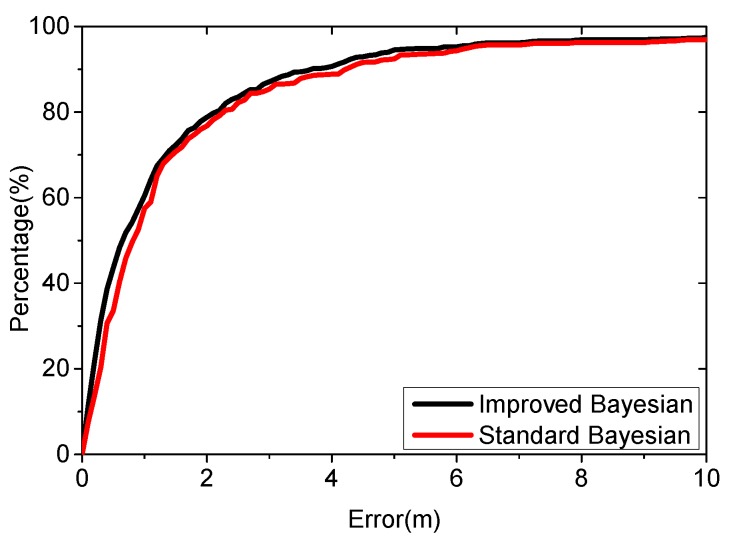
Positioning result using different algorithm.

We make comparison to the standard Bayesian algorithm. Both the new algorithm and the standard algorithm apply the virtual database for positioning. The parameters are set as follows: λ=1, L=7, K=5, ε=0.5. The density of the virtual database is 1. Figure 15 shows the results from 3000 positioning cases.

Figure 15 shows that the new algorithm performs better. The average localization error is 1.84 m using the new algorithm, while the standard is 1.93 m. The new algorithm improves the accuracy by 4.66%, with an average positioning error below 2.2 m for 80% of the cases, while the standard algorithm is 2.3 m.

**Figure 16:**
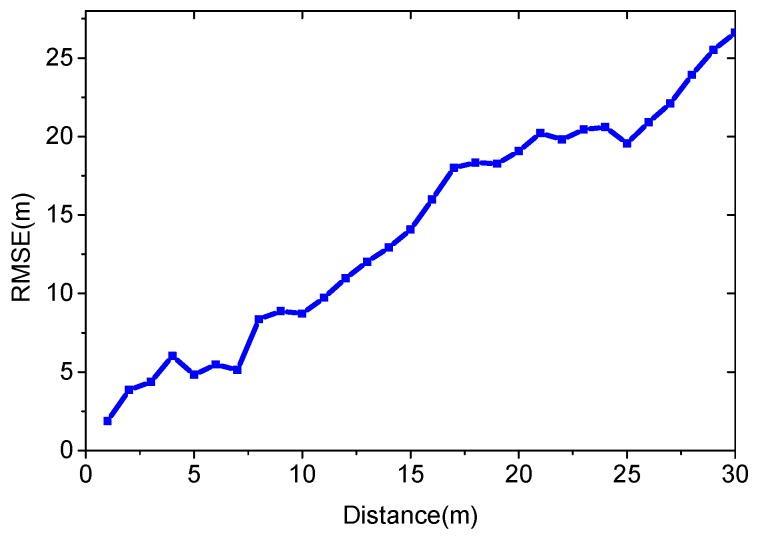
Positioning in different unsurveyed areas using the virtual database.

We evaluate the algorithm in the unsurveyed area. The unsurveyed area is separated into several sub-areas according to the distance to the surveyed area. We compare the positioning accuracy in these sub-areas. Figure 16 shows the experimental results.

Figure 16 illustrates that the RMSE increases as the distance to the surveyed area grows. If the users are less than 10 m away from the surveyed area, the average positioning error is 5.75 m. This positioning result is not accurate enough, but sometimes it is useful, especially for areas without site survey.

**Figure 17:**
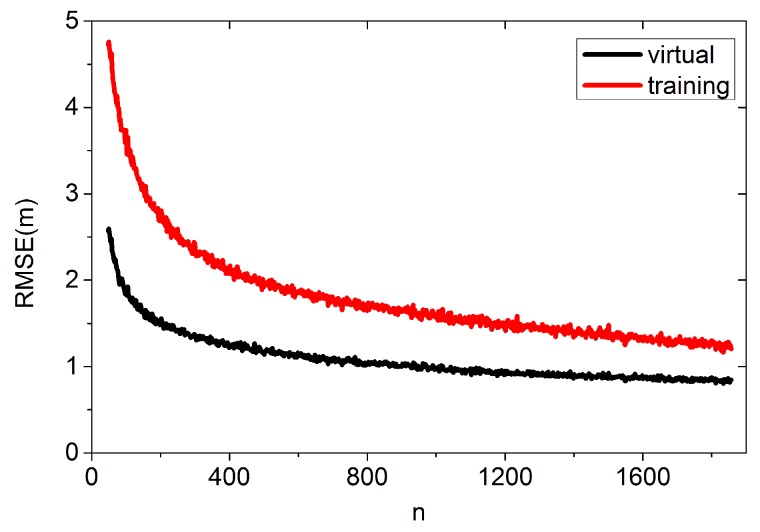
Improving the performance of estimation by crowdsourcing.

The proposed algorithm is scalable, which allows the users to continually upload their coordinates to the server to improve the performance of estimation. Figure 17 shows the experimental result. In this experiment, we use the same brand of smartphone for positioning because different devices report network measurement very differently [37].

In Figure 17, we can see that the proposed algorithm performs better. As the users continually upload their coordinates and RSS measurement, the new algorithm’s performance can be improved.

## 5. Conclusions

The wireless fingerprint technique has the advantages of low deployment cost, supplying reasonable accuracy, and ease of application to mobile devices. As a result, fingerprinting has attracted a lot of attention. In areas without calibration data, however, this algorithm breaks down. Constructing a fingerprint database with high density fingerprint samples is labor-intensive or impossible in some cases. Researchers are continually searching for better algorithms to create and update dense databases more efficiently.

The popularization of smartphones makes mobile crowdsourcing fingerprint localization more practical. However, designing a sustainable incentive mechanism of crowdsourcing remains a challenge. In this paper, we propose a scalable algorithm to create a WLAN radio map by mobile crowdsourcing and Gaussian Process for fingerprint indoor localization. We first propose a Minimum Inverse Distance (MID) algorithm to build a virtual database with uniformly distributed virtual Reference Points (RP). The area covered by the virtual RPs can be larger than the area covered by the training data. A Local Gaussian Process (LGP) is then applied to estimate the virtual RPs’ RSSI values based on the crowdsourced training data. Finally, we improve the Bayesian algorithm to estimate the user’s location using the virtual database.

The parameters in the proposed algorithm are optimized by simulations and the new algorithm is tested on real-case scenarios. The average localization error is 2.47 m using the initial database, while the error in the virtual database is 1.84 m. The new algorithm improves the accuracy by 25.5%, with an average positioning error below 2.2 m for 80% of the cases, while the virtual database is 3.1 m. The proposed algorithm also allows the users to continually upload their coordinates to the server to improve the performance of estimation. Moreover, the proposed algorithm can localize the users in the neighboring unsurveyed area. If the users are less than 10 m away from the surveyed area, the average positioning error is 5.75 m.

The proposed algorithm has to rely on a location server. If there is no connection between the server and clients, the user can’t upload the positioning requirement. As a result, the client won’t receive his coordinate, and this is the problem for all the crowdsourcing fingerprint indoor localization algorithms. Client-based architecture solutions would be more practical. However, with the wide availability of 802.11 WLAN networks, connecting to the internet would be easier. We believe this problem will be solved with the wide deployment of WiFi access points in the future.

Our study requires a strong user collaboration. If the user wants to contribute to the fingerprint database, he should estimate his location with another positioning system and upload the fingerprint, containing the coordinate and the RSS values, to the server. This would lead to the problem that the users are not willing to submit their measurement. For this drawback, we can make the client upload the RSS and location to the server automatically, but the scale of the fingerprint database will increase rapidly. And we are not sure about the reliability of the uploaded data. In that case, we have to filter out the unreliable data. Moreover, we haven’t focused on the device diversity problem. It is practically impossible for all the users to have the same brand of smartphone. Our future work will concentrate on these two issues.

## Figures and Tables

**Table 1:** variance for different algorithms in different areas (dBm) SA: Surveyed Area; UA: Unsurveyed Area; TA: Target Area.

Algorithm	SA	UA	TA
GP	1.86	8.09	5.77
LGP	1.88	8.25	5.88
LDPL	6.81	14.53	7.43
